# Enzymatic synthesis of geranic acid and its protective effects against UVB-induced skin photodamage

**DOI:** 10.3389/fbioe.2026.1891436

**Published:** 2026-10-13

**Authors:** Jiaxin Lin, Ping Zhang, Xianzhe Wang, Lingling Xuan, Shuling Zhang, Jing Wang, Hu Huang

**Affiliations:** PROYA Cosmetics Co., Ltd., Hangzhou, China

**Keywords:** enzymatic synthesis, geranial dehydrogenase, geranic acid, oxidative stress, photodamage

## Abstract

**Background:**

Geranic acid (GA, E−3,7-dimethyl-2,6-octadienoic acid) is a natural monoterpenoid with antimicrobial,anti-inflammatory and antioxidant properties, holding great potential in cosmetics and pharmaceuticals. However, current chemical synthetic routes suffer from poor stereoselectivity and reliance on harsh reagents, restricting its scalable production. This study established a sustainable and stereoselective enzymatic route for producing high-purity GA from citral, and evaluated its protective effect against UVB-induced photodamage in keratinocytes.

**Methods:**

A novel geranial dehydrogenase (GeDH) from *Pseudomonas* sp. was heterologously expressed in *Escherichia coli* BL21 (DE3) via high-density fed-batch fermentation. GA was biosynthesized from citral using a GeDH/NADH oxidase (NOX) cofactor regeneration system, and characterized by GC-QTOF-MS and ^1^H NMR spectroscopy. The anti-photodamage activity and underlying mechanisms of GA were systematically investigated in UVB-irradiated HaCaT keratinocytes.

**Results:**

The biocatalytic system achieved efficient citral-to-GA conversion with over 98% *trans*-stereoisomeric purity, and NOX-mediated cofactor recycling significantly improved process feasibility and economy. In UVB-exposed HaCaT cells, GA dose-dependently alleviated oxidative stress, restored mitochondrial homeostasis, and suppressed Ca^2+^ influx and pro-inflammatory cytokine secretion via downregulating the TRPV1/NF-κB axis. Furthermore, GA attenuated DNA damage, and inhibited the expression of senescence-associated proteins.

**Conclusion:**

This study provides a sustainable biomanufacturing strategy for high-purity GA. The biosynthesized GA exerts multi-layered photoprotection in keratinocytes, strongly supporting its development as a natural anti-photodamage active ingredient for cosmetic and dermatological applications.

## 1 Introduction

Geranic acid (GA, E−3,7-dimethyl-2,6-octadienoic acid) is a naturally occurring monoterpenoid widely applied in fragrances, cosmetics, and pharmaceuticals (Shafeeq et al., 2024). Its ester derivatives are widely used as flavoring and fragrance agents. GA has also been identified as a potential depigmenting agent due to its ability to inhibit tyrosinase activity (Choi, 2012). Direct plant extraction is limited by low natural abundance and variable composition. Consequently, the commercial supply of GA relies heavily on chemical synthesis, typically through the oxidation of geraniol or citral. However, conventional chemical synthetic routes lack stereoselectivity, yielding a mixture of cis (Z) and trans (E) isomers along with impurities such as nerol and citronellol**.** Notably, the trans-isomer exhibits substantially higher biological activity than its cis counterpart (Masuda et al., 2008). Moreover, chemical synthesis and isomer separation involves harsh reagents and generates toxic byproducts, conflicting with green chemistry principles.

Enzymatic biocatalysis represents a promising alternative, providing high regio- and stereoselectivity under mild, eco-friendly conditions (Bayer et al., 2025). In biological systems, GA is generated through the NAD^+^-dependent oxidation of geranial, a reaction catalyzed by geranial dehydrogenase (GeDH) (Wolken and van der Werf, 2001), and preceded by geraniol dehydrogenase-mediated conversion of geraniol to geranial (Luddeke et al., 2012; Shafeeq et al., 2024). Critically, NAD^+^ acts as an essential redox cofactor in these reactions. Since intracellular NAD^+^ is constantly catabolized and must be efficiently recycled to maintain metabolic homeostasis, the success of this biotransformation is often constrained by cofactor availability and the efficiency of the enzymatic cascade (Covarrubias et al., 2021). Despite the identification of GeDHs in certain plants and microbes, their industrial application has been hindered by low expression yields and limited catalytic efficiency, which are often insufficient to effectively drive the NAD^+^ regeneration cycle (Kikonyogo et al., 1999; Muraguchi et al., 2011; Luddeke et al., 2012; Amna et al., 2024). Recent advances in recombinant DNA technology now enable the development of engineered biocatalysts capable of overcoming these thermodynamic and kinetic limitations for monoterpenoid synthesis (Yang et al., 2025).

Beyond the synthesis challenge, the biological function of GA in skin health remains underexplored. Ultraviolet B (UVB) radiation is a primary environmental driver of skin photodamage, triggering ROS overproduction, DNA damage, and inflammation in keratinocytes (Hung et al., 2017). While GA is known to possess antioxidant and anti-inflammatory activities, its protective efficacy and underlying mechanisms against UVB-induced skin photodamage have not been investigated. Regulatory authorities have classified GA as a safe flavoring substance with low acute toxicity, yet its dermatological and photoprotective applications lack formal evaluation, leaving a critical gap for its cosmeceutical development (Adams et al., 2011).

To address these gaps, this study pursued two objectives: (i) developing a recombinant GeDH-NOX biocatalytic system for stereoselective trans-GA synthesis; and (ii) evaluating GA’s protective mechanisms against UVB-induced photodamage in HaCaT cells keratinocytes. This work provides a sustainable GA synthesis route while bridging the gap between GA’s regulatory status and its potential as an anti-photodamage ingredient.

## 2 Materials and methods

### 2.1 Screening of GeDH sequences

A previously reported geranial dehydrogenase (GenBank accession: WP_043683932) was used as a probe to screen for homologous enzymes sharing 30%–90% sequence identity. Homology searches were conducted in Biocyc (https://biocyc.org/), NCBI (https://www.ncbi.nlm.nih.gov/), Brenda (https://www.brenda-enzymes.org/all_enzymes.php), and UniProt (https://www.uniprot.org/).

### 2.2 Expression of GeDH

The GeDH encoding gene was synthesized by Tsingke Biotechnology Co., Ltd. (China), codon-optimized for expression in *E. coli* BL21 (DE3) (TransGen, China), and cloned into the pET-28a (+) expression vector. The resulting recombinant plasmid (50–100 ng) was then transformed into *Escherichia coli* BL21 (DE3) competent cells. Specifically, cells were thawed on ice, mixed with the plasmid, incubated on ice for 30 min, heat-shocked at 42 °C for 90 s, and chilled on ice for 2 min. Subsequently, 900 μL of LB broth was added, and the cells were cultured at 37 °C with shaking at 200 rpm for 1 h. The culture was centrifuged at 1,500 *× g* for 5 min, the supernatant partially removed, and the cell pellet resuspended. The suspension was plated onto LB agar containing 50 mg/L kanamycin sulfate (BBI Solutions, United Kingdom) and incubated inverted at 37 °C for 12 h. Single colonies were verified by PCR and DNA sequencing. Correct clones were inoculated into 50 mL of LB medium (50 mg/L kanamycin) and grown at 37 °C with shaking at 200 rpm. Following centrifugation (5,000 rpm, 4 °C), the cell pellet was resuspended in 5 mL of LB broth mixed with 5 mL of 50% glycerol and stored at −80 °C.

### 2.3 High-density fermentation of engineered strain

A method similar to that reported by Chan et al. was adopted with modifications (Chan et al., 2018). Specifically, Fed-batch fermentation was performed using *E. coli* BL21 (DE3) cells harboring the recombinant GeDH. A glycerol stock (100 μL) was spread onto LB agar plates and incubated at 37 °C for 12 h. The resulting bacterial lawn was inoculated into 100 mL of LB seed medium in a 250 mL baffled flask and cultured at 37 °C with shaking at 200 rpm for 10 h. Subsequently, 200 μL of the seed culture was transferred into 2.6 L of fermentation medium in a 10 L glass bioreactor (Huike NGF-10L, China). The fermentation medium contained (per liter): 1.73 g (NH_4_)_2_SO_4_, 6.72 g KH_2_PO_4_, 9.43 g K_2_HPO_4_, 9 g glucose (Sigma-Aldrich, United States), 1.48 g tryptone (Oxoid, United States), and one drop of antifoam. The pH was maintained at 7.0 using ammonia solution. Aeration was set at 220 L/h air, and agitation was controlled between 500 and 900 rpm. After 15 h of cultivation at 37 °C and 600 rpm, continuous feeding was initiated with a feed solution containing 195 g/L glucose (Sigma-Aldrich, United States), 32 g/L tryptone (Oxoid), 32 g/L yeast extract (Oxoid), and 60 mM MgSO_4_ (Macklin, China). Cultivation continued until the OD600 reached 30–40, at which point 0.1 mM isopropyl-β-D-thiogalactopyranoside (IPTG; Sangon Biotech, China) was added, and induction was carried out at 30 °C for 6 h. Finally, the cells were harvested and disrupted by high-pressure homogenization to obtain the crude enzyme extract.

### 2.4 Enzymatic synthesis system for GA production

The enzymatic conversion of citral (an isomeric mixture of geranial and neral) to GA was catalyzed by cell lysate supernatant containing GeDH, with NAD^+^ serving as the essential cofactor. The optimized 300 mL reaction system contained: 2 mM NAD^+^, 6 mL citral, 244 mL cell lysate containing GeDH, and 50 mL cell lysate containing NADH oxidase (NOX), in 100 mM Na_2_HPO_4_-NaH_2_PO_4_ buffer (pH 8.0). The reaction was carried out at 30 °C, 200 rpm for 12 h. Samples at 0 and 12 h were extracted with ethyl acetate (1:1 v/v; Macklin) for 1 h, and the organic phase was analyzed by gas chromatography (GC) for residual citral and produced GA.

### 2.5 Gas chromatography (GC) analysis

The reaction products were analyzed by GC using a Fuli 9790II instrument equipped with an FID detector and a Kromat KB-624 capillary column (50 m × 0.3 mm × 1 μm). N_2_ served as the carrier gas. A 2 μL sample was injected at a split ratio of 1:50, with the injector and detector both maintained at 300 °C. The column temperature was held isothermally at 220 °C for 15 min.

### 2.6 Purification of GA

The enzymatic reaction broth (300 mL) was extracted twice with an equal volume of ethyl acetate. The combined organic phase was separated by centrifugation at 6,000 rpm for 10 min, and the supernatant was collected. The organic phase was dried over anhydrous sodium sulfate to remove residual water, filtered, and concentrated under reduced pressure at 40 °C. The resulting crude extract was mixed with 300–400 mesh silica gel and rotary evaporated to dryness for dry loading. Column chromatography was performed on a silica gel column, eluted with a petroleum ether–ethyl acetate mixture (5:1, v/v). Fractions were collected and monitored by thin-layer chromatography (TLC) using the same solvent system. The target compound GA was identified by comparison of its Rf value with that of an authentic standard, and the corresponding fractions were pooled, concentrated, and used for downstream assays.

### 2.7 Gas chromatography with time-of-flight mass spectrometry (GC-QTOF-MS) analysis

The GC-QTOF-MS was employed to evaluate the purity of the obtained GA. The GA sample (0.1 μL) was injected in split mode (20:1) into a GC-QTOF system (Agilent, United States). Chromatographic separation was achieved on an Agilent HP-5MS UI capillary column (15 m × 250 μm, 0.25 μm). High-purity helium (He) was utilized as the carrier gas under constant flow conditions at a rate of 1.2 mL/min. The oven temperature program was programmed as follows: an initial hold at 50 °C for 3 min, then raised to 300 °C at a rate of 15 °C/min, held for 5 min. The total analysis time was 24.667 min. Mass spectrometric detection was performed using an electron ionization (EI) source operated at 70 eV. The transfer line and ion source temperatures were set to 250 °C and 230 °C, respectively. Data were acquired over a mass range of 50–475 m/z in full-scan total ion current (TIC) mode, with a fragmentation voltage of 70.0 V applied to ensure robust molecular fingerprinting. Post-acquisition, mass spectra were identified and confirmed by matching against the NIST mass spectral library.

### 2.8 Cell culture and treatment

HaCaT human immortalized keratinocytes were purchased from Shanghai Cell Bank of the Chinese Academy of Sciences (China). Cells were cultured in DMEM high-glucose medium (Gibco, United States) supplemented with 10% fetal bovine serum (Thermo Fisher Scientific, United States) and 1% penicillin–streptomycin (Solarbio, China), at 37 °C in a 5% CO_2_ humidified incubator. Logarithmic-phase cells were used for all experiments. Cell viability was measured by CCK-8 kit (Abbkine, China) according to the manufacturer’s instructions.

For the UVB-induced photodamage model, cells were exposed to a single, precisely calibrated UVB dose of 15 mJ/cm^2^ using a UVB lamp (Bio-Sun UV irradiation system, Vilber Lourmat, France). Immediately after UVB exposure, cells were treated with GA for 24 h at indicated dosage.

### 2.9 Detection of intracellular ROS and mitochondrial membrane potential (ΔΨm)

Intracellular ROS levels were measured using the DCFH-DA fluorescent probe kit (Beyotime, China). Mitochondrial membrane potential (ΔΨm) was determined with a JC-1-based mitochondrial membrane potential assay kit (Beyotime, China) according to the manufacture’s instruction. After treatment, HaCaT cells were incubated with DCFH-DA probe or JC-1 working solution at 37 °C for 20 min and analyzed with a CytoFLEX LX flow cytometer (Beckman Coulter, United States). The data were analyzed with FlowJo_v10.0.8 (TreeStar, United States).

### 2.10 Measurement of oxidative stress markers

HaCaT cells were lysed in RIPA buffer (Beyotime) supplemented with protease inhibitors on ice for 30 min, followed by centrifugation at 12,000 rpm, 4 °C for 5 min. Total protein concentration was quantified using a BCA protein assay kit (Thermo Fisher Scientific, United States). Malondialdehyde (MDA) content, superoxide dismutase (SOD) and glutathione peroxidase (GPx) activities were measured using commercial kits (Beyotime, China) according to the manufacturer’s instructions.

### 2.11 Enzyme-linked immunosorbent assay (ELISA)

After indicated treatment, cell culture supernatants were collected and centrifuged at 3,000 rpm for 10 min. The secretion levels of monocyte chemoattractant protein-1 (MCP-1), interleukin-6 (IL-6), matrix metalloproteinase-1 (MMP-1), and MMP-3 were quantified with corresponding ELISA kits (Beyotime) according to the manufacture’s instruction. The optical density at 450 nm (OD450) was measured using a FlexStation 3 microplate reader (Molecular Devices, United States).

### 2.12 Immunofluorescence analysis

HaCaT cells were fixed with 4% paraformaldehyde for 15 min, permeabilized with 0.1% Triton X-100 for 20 min, and blocked with 1% BSA for 1 h. Cells were incubated with primary antibodies (1:200 dilution) overnight at 4 °C, followed by fluorescent secondary antibodies (1:1,000 dilution) for 2 h at room temperature. Nuclei were stained with 1 μg/mL DAPI for 5 min. Images were captured using a BZ-X710 fluorescence microscope (Keyence, Japan) and analyzed using ImageJ 2.0 (National Institutes of Health, United States) software. The primary antibodies used were NF-κB p65 rabbit polyclonal antibody (AF0246), TRPV1 rabbit polyclonal antibody (AF8250), and phospho-histone H2AX (Ser139) rabbit polyclonal antibody (AF5836) (Beyotime). Secondary antibodies were BeyoAb™ Cy3-labeled Goat Anti-Rabbit IgG (H + L) (A1623) and BeyoAb™ FITC-labeled Goat Anti-Rabbit IgG (H + L) (A1800) (Beyotime, China).

### 2.13 Detection of intracellular Ca^2+^ levels

Intracellular Ca^2+^ levels were measured using Fluo-4 AM (Beyotime, China). Cells were incubated with 5 μM Fluo-4 AM in Ca^2+^-free HBSS containing 0.02% pluronic F-127 at 37 °C for 30 min. Intracellular Ca^2+^ levels were detected by flow cytometry. The images were analyzed with FlowJo_v10.0.8 (TreeStar).

### 2.14 Western blot

Total protein was extracted from HaCaT cells using pre-chilled lysis buffer (Solarbio, China) and quantified using a BCA protein assay kit (Beyotime, China). Proteins were separated by 12% SDS-PAGE and then transferred onto PVDF membranes. Membranes were blocked with 5% skimmed milk for 1 h, then incubated with primary antibodies overnight at 4 °C. After washing with Tris-buffered saline containing Tween 20 (TBST), HRP-conjugated secondary antibodies (1:1,000 dilution) were applied and incubated for 1 h. Protein bands were visualized using a BioSpectrum imaging system (Ultra-Violet Products Ltd., United Kingdom) and semi-quantified using ImageJ 2.0 software. The primary antibodies used were anti-β-actin (mouse, ab8226), anti-p16 (rabbit, ab108349), and anti-p21 (rabbit, ab188224) (Abcam, United Kingdom). Secondary antibodies were HRP-conjugated anti-mouse IgG (ab6728) or anti-rabbit IgG (ab6721) (Abcam, United Kingdom).

### 2.15 Statistical analysis

All experiments were performed with at least three independent biological replicates, and each biological replicate contained three technical replicates. Data are presented as means ± SD with individual data points shown. Statistical analysis was performed using GraphPad Prism 8.0.2 by one-way ANOVA followed by Tukey’s test for multiple comparisons. Exact P values and 95% confidence intervals are reported in the figure legends.

## 3 Results

### 3.1 Biocatalytic synthesis and characterization of high-purity GA

Following sequence-based screening, a total of ten candidate GeDHs were identified (Supplementary Table S1). Among these candidates, a GeDH derived from *Pseudomonas* sp., designated KDI24_07135, exhibited the highest catalytic activity, and was thus selected for subsequent experiments (Supplementary Table S2). Bioinformatic characterization revealed that KDI24_07135 consists of 478 amino acids, with a predicted molecular mass of 50.57 kDa, a theoretical isoelectric point (pI) of 5.02, and high predicted structural stability.

To achieve sustainable and stereoselective production of GA, the recombinant GeDH was successfully expressed in *E. coli* BL21 (DE3). SDS-PAGE analysis of Ni-NTA affinity-purified recombinant KDI24_07135 showed that the target protein was effectively eluted with 200–300 mM imidazole, verifying the successful heterologous expression and purification of GeDH (Supplementary Figures S1a, S1b).

High-density fed-batch fermentation of the engineered strain yielded a GeDH-containing cell lysate with high catalytic activity. Under optimized reaction conditions (30 °C, pH 8.0, 12 h), the biocatalytic system achieved high-yield conversion of citral to GA. In the 300 mL reaction system containing 20 g/L citral (approximately 131 mM), the conversion yield reached >90%, with a product titer of approximately 15 g/L GA (Figure 1a). And efficient substrate conversion was further confirmed by GC analysis (Figure 1b). The GC-QTOF-MS total ion current chromatogram of the purified product showed a dominant peak at a retention time of 11.05 min, accounting for over 98% of the total ion current, indicating high stereoisomeric purity (Figure 1c). The corresponding mass spectrum displayed a molecular ion peak [M]^+^ at m/z 168, consistent with the molecular formula of GA (C_10_H_16_O_2_) (Figure 1d).

**FIGURE 1:**
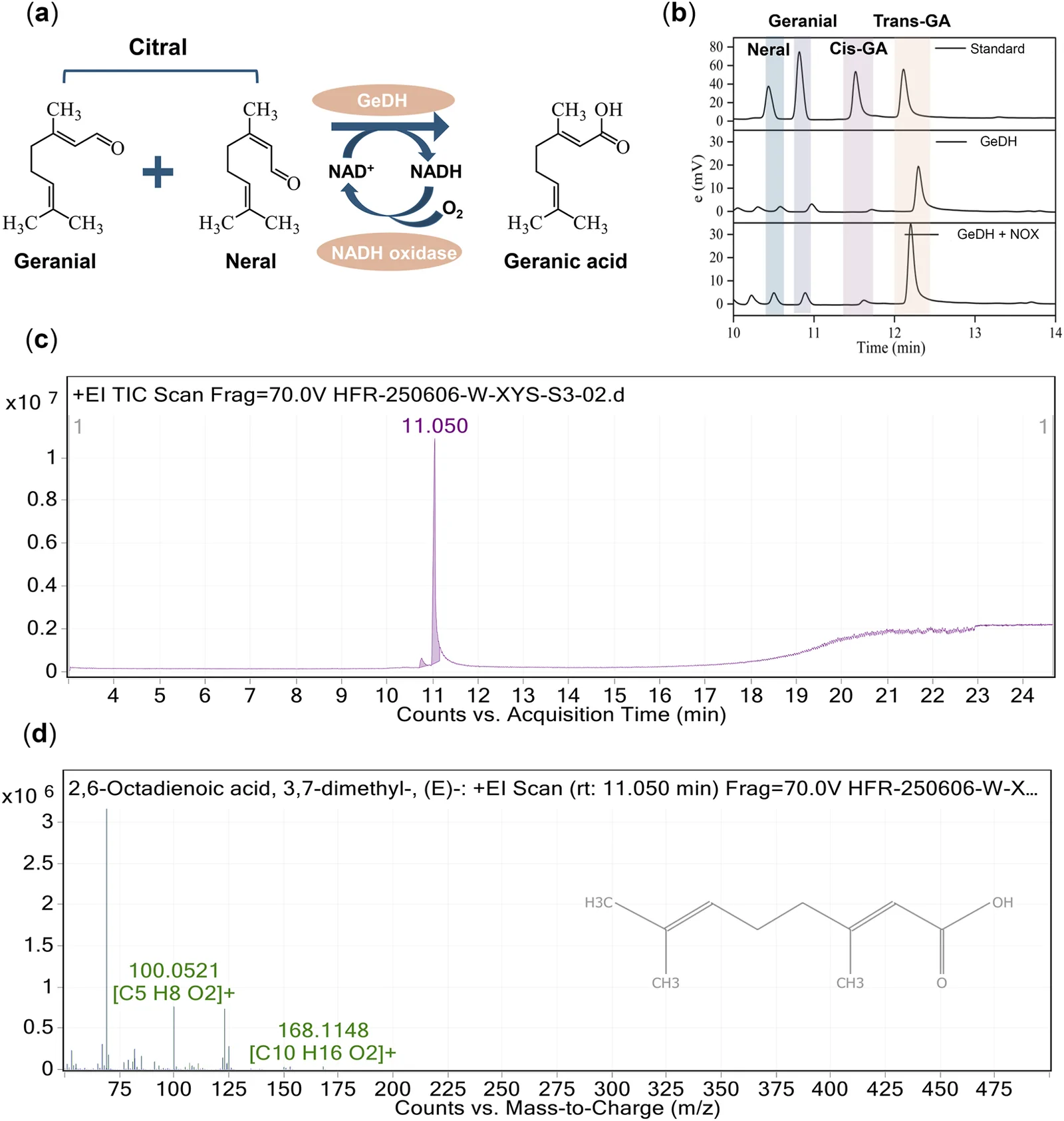
Biocatalytic synthesis and characterization of high-purity geranic acid (GA). **(a)** Schematic of the enzymatic conversion of citral to GA using recombinant GeDH with NAD^+^ and a NOX-based cofactor regeneration system. **(b)** GC analysis of the reaction mixture. **(c)** GC-QTOF-MS total ion current chromatogram of purified GA. **(d)** Mass spectrum of the purified GA.

^1^H NMR spectroscopy further validated the chemical identity and purity of the product. All proton resonance signals were fully assigned, and their chemical shifts, peak shapes, and integral ratios were in good agreement with the reference spectrum of geranic acid. The spectrum exhibited a flat baseline with no resolvable impurity peaks, confirming favorable sample purity (Supplementary Figure S2). Collectively, these results demonstrate the high efficiency and stereoselectivity of the established biocatalytic process.

### 3.2 GA alleviates UVB-induced oxidative stress and mitochondrial dysfunction

We first evaluated the cytotoxicity of GA in HaCaT keratinocytes. GA exhibited no obvious cytotoxicity towards HaCaT cells within the concentration range of 0–250 mg/L, with cell viability remaining above 90% after 24 h of treatment. Accordingly, concentrations of 62.5 mg/L (L-GA), 125 mg/L (M-GA), and 250 mg/L (H-GA) were selected for subsequent experiments (Figure 2a).

**FIGURE 2:**
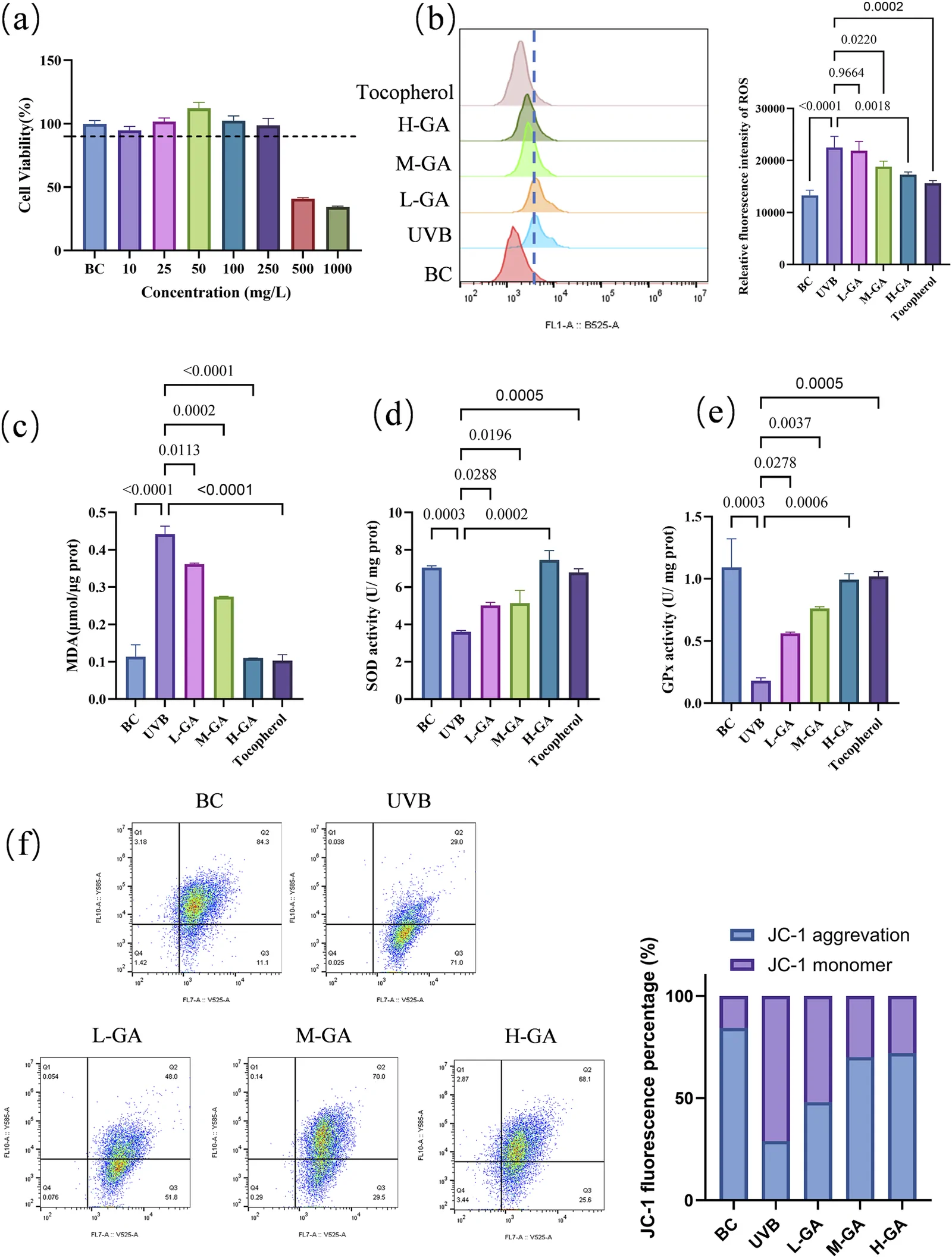
Geranic acid (GA) alleviates UVB-induced oxidative stress and mitochondrial dysfunc-tion in HaCaT keratinocytes. **(a)** Cell viability assay of GA-treated HaCaT cells. **(b)** Flow cytometry analysis of intracellular ROS levels using DCFH-DA staining. **(c–e)** Quantification of MDA levels, SOD activity, and GPx activity. 50 mg/L α-tocopherol was used as the positive control. **(f)** JC-1 staining for mitochondrial membrane potential (ΔΨm).

UVB irradiation induced a substantial elevation in intracellular reactive oxygen species (ROS) levels (Fisher et al., 2002). GA treatment dose-dependently suppressed ROS overproduction (Figure 2b). In line with this finding, UVB exposure markedly increased the content of malondialdehyde (MDA), a core marker of lipid peroxidation, while GA administration significantly reduced MDA accumulation in a dose-dependent manner (Figure 2c). Furthermore, the activities of two key endogenous antioxidant enzymes, superoxide dismutase (SOD) and glutathione peroxidase (GPx), were markedly suppressed by UVB irradiation, and this suppression was effectively reversed by GA treatment across all tested doses (Figures 2d,e). We further assessed mitochondrial function via JC-1 staining. UVB exposure triggered evident mitochondrial membrane potential (ΔΨm) depolarization, as reflected by an increased monomer-to-aggregate fluorescence ratio. GA treatment reversed ΔΨm loss, with medium and high concentrations showing the most prominent protective effects (Figure 2f).

### 3.3 GA suppresses UVB-induced inflammation and intracellular ca^2+^ influx

UVB radiation is known to trigger inflammatory cascades and disrupt intracellular calcium homeostasis (Wu et al., 2013; Salminen et al., 2022). In this study; we found UVB irradiation significantly upregulated the secretion of the pro-inflammatory mediator MCP-1 and IL-6. GA treatment markedly suppressed the secretion of both cytokines, and the inhibitory effect strengthened with increasing dosage (Figures 3a,b). Additionally, Fluo-4 AM fluorescence detection indicated that UVB exposure induced a massive influx of intracellular Ca^2+^ in HaCaT cells, and this calcium overload was effectively attenuated by GA treatment (Figure 3c). Immunofluorescence analysis corroborated these findings at the protein level. UVB irradiation significantly upregulated the expression of transient receptor potential vanilloid 1 (TRPV1) and nuclear factor kappa-B (NF-κB). GA treatment dose-dependently downregulated both proteins expression, consistent with its inhibitory effects on calcium influx and inflammatory cytokine secretion (Figure 3d).

**FIGURE 3:**
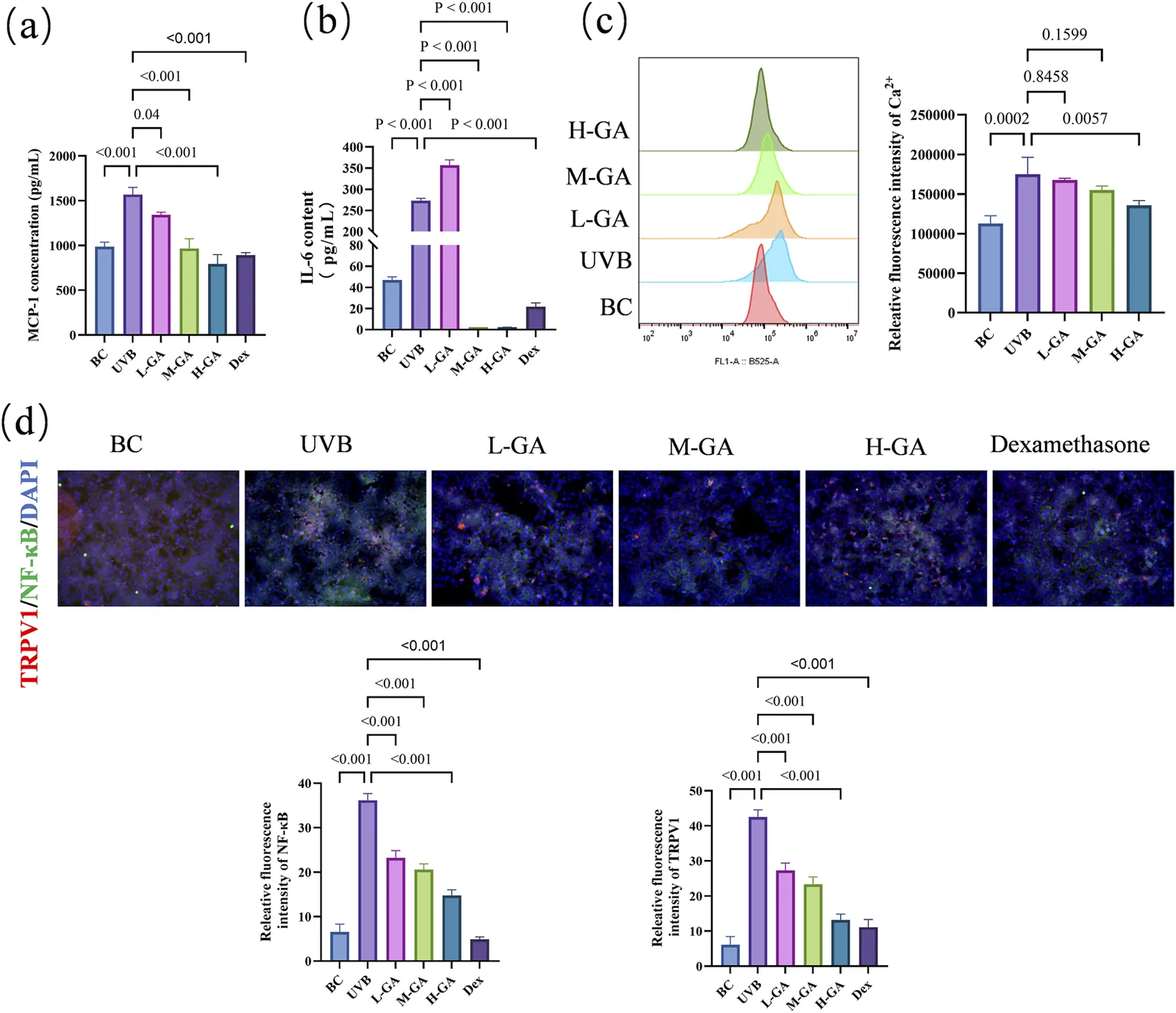
Geranic acid (GA) suppresses UVB-induced inflammation and Ca^2+^ influx. **(a,b)** ELISA quantification of MCP-1 and IL-6 levels. 1 mg/L dexamethasone (Dex) was used as the positive control. **(c)** Flow cytometry analysis of intracellular Ca^2+^ levels using Fluo-4 AM. **(d)** Immunofluorescence staining and quantification of NF-κB and TRPV1 ex-pression (×200 magnification).

### 3.4 GA mitigates UVB-Induced DNA damage and cellular senescence

Persistent oxidative stress and inflammation ultimately drive DNA damage and cellular senescence (Kammeyer and Luiten, 2015). We thus evaluated the protective effects of GA against these endpoints. Immunofluorescence analysis of γ-H2AX foci, a marker of DNA double-strand breaks, showed that UVB irradiation led to significant increase c in γ-H2AX foci formation. GA treatment significantly reduced γ-H2AX accumulation (Figure 4a). Western blot analysis further demonstrated that UVB exposure significantly upregulated the expression of senescence-associated proteins p16 and p21 in keratinocytes, and this upregulation was effectively reversed by GA treatment (Figure 4b). Additionally, UVB irradiation markedly increased the secretion of matrix metalloproteinases MMP-1 and MMP-3, which mediate extracellular matrix degradation during photodamage (Lee et al., 2021). GA treatment dose-dependently attenuated the upregulation of both MMPs, suggesting a protective effect against UVB-induced extracellular matrix damage (Figure 4c).

**FIGURE 4:**
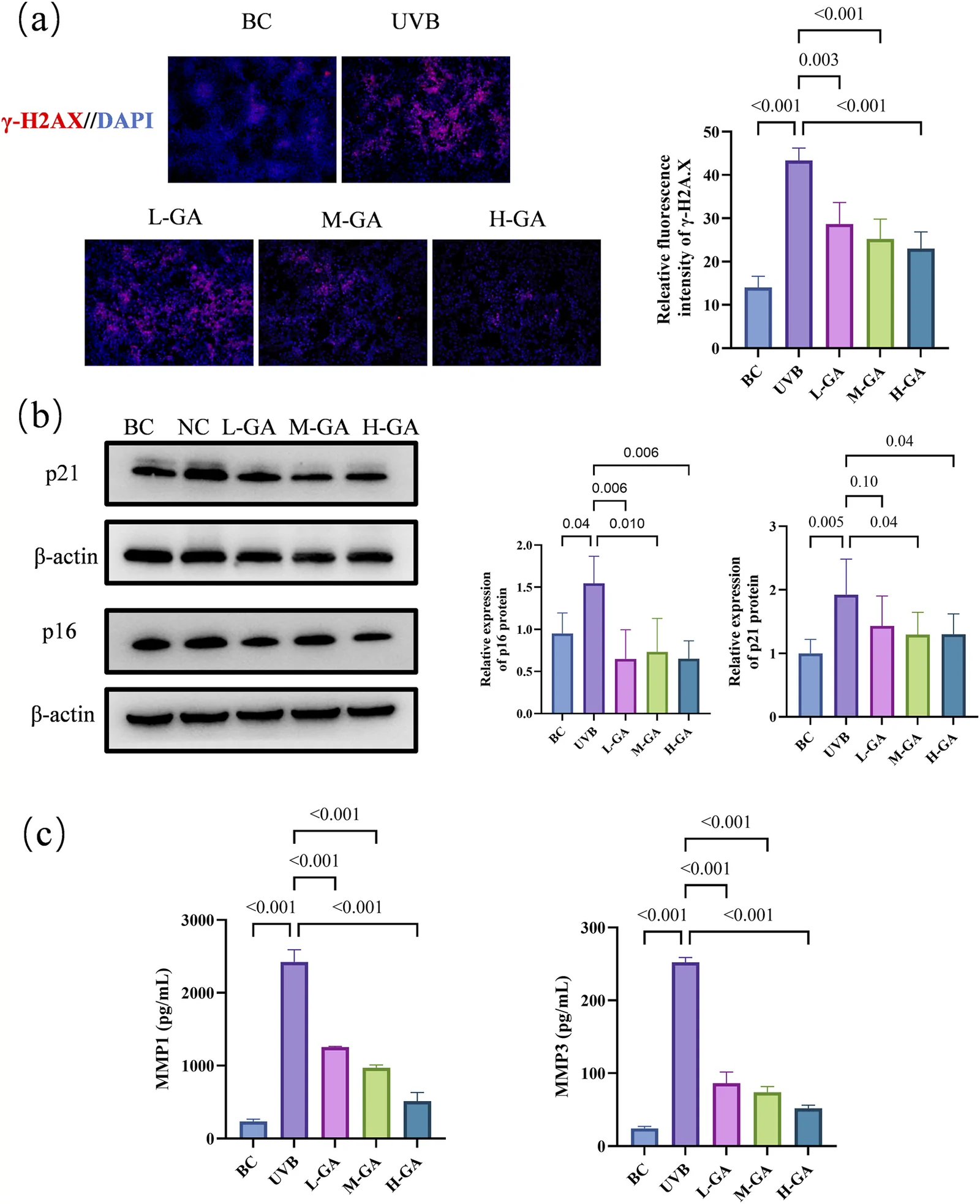
Geranic acid (GA) reduces UVB-induced DNA damage and cellular senescence. **(a)** Immunofluorescence staining and quantification of γ-H2AX foci (×200 magnification). **(b)** Western blot analysis and quantification of p16 and p21 expression. **(c)** ELISA quantification of MMP-1 and MMP-3 levels.

## 4 Discussion

GA is a naturally occurring monoterpenoid with documented antimicrobial and anti-inflammatory properties (Mi et al., 2014), yet its potential in skin photoprotection and sustainable industrial production remain largely underexplored. This study developed a recombinant GeDH/NOX biocatalytic system for stereoselective high-purity *trans*-GA synthesis, and systematically elucidated its protective mechanisms against UVB-induced photodamage in HaCaT keratinocytes.

The biocatalytic strategy employed in this study addresses several key limitations of conventional chemical synthesis of GA. Traditional citral oxidation routes lack stereoselectivity, produce *E*/*Z* isomer mixtures and toxic byproducts, and require laborious downstream purification to obtain bioactive *trans*-GA (Anand et al., 2025). In contrast, our GeDH-based system achieves one-step stereoselective oxidation under mild aqueous conditions, delivering *trans*-GA with over 98% isomeric purity. The NOX-mediated cofactor regeneration system employs oxygen as the terminal electron acceptor and generates only water as a byproduct, conforming to green chemistry principles and reducing purification burdens. Compared to previously reported aldehyde dehydrogenases from other bacterial sources (Luddeke et al., 2012), our GeDH candidate (KDI24_07135), derived from *Castellaniella defragrans,* showed high expression yield and catalytic efficiency, likely due to codon optimization and fed-batch fermentation. These results position the enzymatic route as a promising green alternative for GA production.

This high stereoselectivity is particularly important because the trans (E) isomer of GA exhibits superior biological activity compared to the cis (Z) form (Masuda et al., 2008). In our study, at the cellular level, we found GA exerts protective effects against UVB-induced photodamage. UVB radiation is a major environmental trigger of skin photodamage, stimulating keratinocytes to overproduce ROS, which induces DNA photodamage and activates MAPK and NF-κB signaling, leading to MMP upregulation, extracellular matrix degradation, inflammation, and cellular senescence (Liao et al., 2026). Our results demonstrated that GA treatment dose-dependently reduced intracellular ROS levels and restored the activities of key antioxidant enzymes SOD and GPx. These findings suggest that GA not only directly scavenges ROS but also enhances the endogenous antioxidant defense system. The observed restoration of ΔΨm further supports a protective role of GA at the organelle level, as mitochondrial dysfunction is both a cause and a consequence of oxidative stress (Liao et al., 2026). Additionally, we investigated the regulatory effect of GA on UVB-induced calcium signaling and inflammation. UVB exposure triggered a marked influx of intracellular Ca^2+^ and upregulation of TRPV1, a calcium-permeable ion channel implicated in skin inflammation and pain (Vangeel and Voets, 2019; Hsu et al., 2020). GA treatment effectively blocked Ca^2+^ influx, reduced TRPV1 expression, and suppressed NF-κB expression (Yoon et al., 2025), which is consistent with the reduced secretion of pro-inflammatory cytokines IL-6 and MCP-1.

Moreover, GA treatment significantly reduced UVB-induced DNA damage, as evidenced by decreased γ-H2AX foci formation, a sensitive marker of DNA double-strand breaks (Mah et al., 2010). And downregulates senescence-associated proteins p16 and p21. Additionally, GA inhibits secretion of matrix metalloproteinases MMP-1 and MMP-3, which mediate extracellular matrix degradation in photodamage (Lee et al., 2021). These findings are consistent with the growing interest in natural compounds for photodamage prevention and highlight the translational potential of GA in dermatological applications (Ilma et al., 2025).

Several limitations should be acknowledged. First, although the biocatalytic system achieved high purity, substrate concentrations were restricted to prevent enzyme inhibition. Second, all biological evaluations were conducted *in vitro* in HaCaT cells, requiring further validation in animal models and human skin tissue. Additionally, although the anti-inflammatory and antioxidant activities of GA have been widely reported, the molecular basis underlying GA-mediated modulation of the Ca^2+^/TRPV1/NF-κB signaling axis in skin photodamage models remains to be further elucidated.

## 5 Conclusion

This study established a green biocatalytic system based on the GeDH/NOX cofactor recycling system, which achieved the stereoselective synthesis of high-purity trans-geranic acid (>98% stereoisomeric purity) from citral, effectively addressing the poor stereoselectivity and environmental unfriendliness of traditional chemical synthesis. The biosynthesized GA exhibited potent anti-UVB photodamage effects in HaCaT keratinocytes via multiple mechanisms, including alleviating oxidative stress, maintaining mitochondrial homeostasis, inhibiting the Ca^2+^/TRPV1/NF-κB inflammatory cascade, and attenuating DNA damage and cellular senescence. These findings provide mechanistic foundations and production technical support for the application of GA as a promising natural anti-photodamage ingredient.

## Data Availability

The original contributions presented in the study are included in the article/Supplementary Material, further inquiries can be directed to the corresponding author.
