# Supplementary material for: Enzymatic synthesis of geranic acid and its protective effects against UVB-induced skin photodamage
Source: Front Bioeng Biotechnol. 2026 Oct 13;14:1891436. doi: 10.3389/fbioe.2026.1891436 (PMC13710334; doi:10.3389/fbioe.2026.1891436)
Supplement: Supplementary file 1 [file Table1.docx]

Table S1 Basic Information of Mined Geranial Dehydrogenas

| Geranial dehydrogenase | Source | Number of amino acids | Mw (kDa) | pI | stability |
| --- | --- | --- | --- | --- | --- |
| WP_043683932 | *Castellaniella defragrans* | 478 | 50.64 | 5.42 | Unstable |
| Br6_04489 | *Rhodococcus sp. Br-6* | 487 | 51.26 | 5.16 | Unstable |
| KDI24_07135 | *Pseudomonadales bacterium* | 478 | 50.57 | 5.02 | Stable |
| AldA-Rv0768 | *Thauera terpenica* | 478 | 50.82 | 5.4 | Unstable |
| CFAEC_02300 | *Corynebacterium faecale* | 502 | 52.90 | 4.75 | Unstable |
| JSS57_06825 | *Proteobacteria bacterium* | 478 | 50.49 | 5.25 | Stable |
| AAGA71_06380 | *Pseudomonadota bacterium* | 477 | 50.65 | 4.93 | Stable |
| SMF913_12953 | *Streptomyces malaysiensis* | 488 | 51.91 | 4.99 | Unstable |
| NTW01_04870 | *Gammaproteobacteria bacterium* | 483 | 50.91 | 5.7 | Stable |
| AAF724_03170 | *Pseudomonadota bacterium* | 477 | 50.76 | 4.97 | Stable |

**Analysis of Geranial Dehydrogenase Enzyme Activity**

The enzymatic activity of geranial dehydrogenase was determined via spectrophotometry. Geranial dehydrogenase is an NAD⁺-dependent enzyme that catalyses the reduction of NAD⁺ to NADH during the catalytic reaction. Since NADH has characteristic ultraviolet absorbance at 340 nm, the activity of geranial dehydrogenase was quantified by monitoring the time-dependent increase in absorbance at OD₃₄₀.

The enzymatic reaction mixture (total volume of 200 μL) consisted of 5 μL enzyme sample, substrate (citral or geranial, final concentration 1 mM), and NAD⁺ (final concentration 1 mM). The system was brought to a final volume using 100 mM Na₂HPO₄–NaH₂PO₄ buffer (pH 8.0). The reaction was performed at 30°C, and the absorbance variation at 340 nm was continuously measured using a multi-mode microplate reader. One unit (U) of enzyme activity was defined as the quantity of enzyme that catalyses the reduction of 1 μmol NAD⁺ per minute under the assay conditions.

Enzyme Activity (U) = $\frac{EW\times V\times{10}^{3}}{6220\times L\times v}$ ,

EW, change in absorbance within 1 min; 6220, extinction coefficient, L/(mol·cm); V, reaction volume, mL; v, volume of enzyme added, mL; L, optical path length, cm.

Table S2 Determination of Geranial Dehydrogenase Activity

| symbol | Protein | Enzyme activity (U/mL)^a^ | Enzyme activity (U/mL)^b^ | Enzyme activity (U/mL)^c^ | Enzyme activity (U/mL)^d^ |
| --- | --- | --- | --- | --- | --- |
| C | pET28a | ND^e^ | ND^e^ | ND^e^ | ND^e^ |
| 1 | pET28a-*WP_043683932* | 0.22±0.02 | 0.54±0.06 | 0.21±0.02 | 0.32±0.03 |
| 2 | pET28a-*Br6_04489* | ND^e^ | ND^e^ | ND^e^ | ND^e^ |
| 3 | pET28a-*KD124_07135* | 0.80±0.06 | 1.46±0.11 | 0.40±0.03 | 0.28±0.03 |
| 4 | pET28a-*AldA_Rv0768* | 0.34±0.01 | 0.66±0.11 | 0.14±0.05 | 0.17±0.02 |
| 5 | pET28a-*CFAEC_02300* | ND^e^ | ND^e^ | ND^e^ | ND^e^ |
| 6 | pET28a-*JSS57_06825* | 0.53±0.05 | 0.69±0.07 | 0.03±0.00 | 0.02±0.00 |
| 7 | pET28a-*AAGA71_06380* | ND^e^ | ND^e^ | ND^e^ | ND^e^ |
| 8 | pET28a-*SMF913_12953* | ND^e^ | ND^e^ | ND^e^ | ND^e^ |
| 9 | pET28a-*NTW01_04870* | ND^e^ | ND^e^ | ND^e^ | ND^e^ |
| 10 | pET28a-*AAF724_03170* | ND^e^ | ND^e^ | ND^e^ | ND^e^ |

a: The substrate is citral, and the protein incubation assay is performed in 100 mM Na₂HPO₄-NaH₂PO₄ buffer at pH 8.0

b: The substrate is geranial, and the protein incubation assay is performed in 100 mM Na₂HPO₄-NaH₂PO₄ buffer at pH 8.0

c: The substrate is citral, and the protein incubation assay is performed in 100 mM Glycine-NaOH buffer at pH 9.4

d: The substrate is geranial, and the protein incubation assay is performed in 100 mM Glycine-NaOH buffer at pH 9.4

e: ND indicates no detectable activity

Figure S1(a). SDS-PAGE analysis of geranial dehydrogenase expression

M, Protein Marker ranging from 18 to 66 kDa; C, cell lysate and supernatant of pET28a expressed in BL21; 1, cell lysate and supernatant of pET28a-WP_043683932 expressed in BL21; 2, cell lysate and supernatant of pET28a-Br6_04489 expressed in BL21; 3, cell lysate and supernatant of pET28a-KD124_07135 expressed in BL21; 4, cell lysate and supernatant of pET28a-AldA_Rv0768 expressed in BL21; 5, cell lysate and supernatant of pET28a-AldA_Rv0768 expressed in BL21; 6, cell lysate and supernatant of pET28a-JSS57_06825 expressed in BL21; 7, cell lysate and supernatant of pET28a-AAGA71_06380 expressed in BL21; 8, cell lysate and supernatant of pET28a-SMF913_12953 expressed in BL21; 9, cell lysate and supernatant of pET28a-NTW01_04870 expressed in BL21; 10, cell lysate and supernatant of pET28a-AAF724_03170 expressed in BL21

Figure S1(b). SDS-PAGE analysis of the Ni-NTA affinity purification of recombinant KDI24_07135.

Lanes: M, protein molecular weight marker; Cell lysate, clarified E. coli cell lysate; Flow-through, unbound fraction after loading onto Ni-NTA resin; Eluate, fractions eluted sequentially with 50, 100, 200, 300 and 500 mM imidazole. The target protein KDI24_07135 was predominantly eluted with 200–300 mM imidazole.

Figure S2. ¹H NMR spectrum of geranic acid (GA). NMR measurement was performed in CDCl₃ at 400 MHz. Chemical shifts (δ) are given in ppm. All characteristic proton signals of geranic acid were assigned, and no obvious impurity peaks were observed.
